# Determination of Particle Size of Active Pharmaceutical Ingredients in Dry Powder Inhaler Formulations

**DOI:** 10.3390/ph19040543

**Published:** 2026-03-28

**Authors:** Stefani Fertaki, Malvina Orkoula, Christos Kontoyannis

**Affiliations:** 1 Department of Pharmacy, University of Patras, GR-26504 Patras, Greece; sfertaki@iceht.forth.gr (S.F.); malbie@upatras.gr (M.O.); 2 Institute of Chemical Engineering Sciences, Foundation of Research and Technology-Hellas (ICE-HT/FORTH), GR-26504 Platani, Achaias, Greece

**Keywords:** particle size, dry powder inhalers, active ingredients, micro-Raman spectroscopy, scanning electron microscopy, energy-dispersive X-ray microanalysis

## Abstract

**Background/Objectives:** Accurate determination of active pharmaceutical ingredient (API) particle size within dry powder inhaler (DPI) formulations is essential for ensuring effective pulmonary delivery but remains analytically challenging due to low API content and micronized particle size. **Methods:** In this study, scanning electron microscopy (SEM) coupled with energy-dispersive X-ray microanalysis (EDX) was used to directly identify and calculate the API particle size within several different commercial DPI products fit for purpose under regulatory constraints. The method exploits unique elemental markers inherent to each API, enabling reliable discrimination from excipients without prior sample modification or API extraction. **Results:** Large-area SEM–EDX mapping was used to localize API particles, followed by high-magnification imaging and confirmatory spot microanalysis. Particle sizes were manually measured for at least 50 API particles per formulation using image analysis software, and particle size distribution parameters were calculated from equivalent spherical diameters. **Conclusions:** The methodology was successfully applied to Spiriva^®^, Anoro^®^ Ellipta, and Relvar^®^ Ellipta inhalation powders, revealing micronized APIs with distinct morphological features and verifying systematic application across products. Cross-validation against laser diffraction measurements of pure APIs demonstrated statistical equivalence, confirming the robustness and analytical utility of the proposed method for particle size assessment in DPI formulations.

## 1. Introduction

The particle size and morphology of active pharmaceutical ingredients is a major concern in drug development, affecting absorption rate, bioavailability, manufacturability and the overall quality and performance of solid dosage forms [1,2,3,4,5,6,7]. In dry powder inhalers, particle size determination of APIs is particularly important owing to the stringent specifications required for pulmonary delivery. DPI formulations typically consist of an ordered mixture of micronized drug particles and larger lactose carrier particles, the latter being incorporated to enhance powder flowability. The patient’s inhalation through the device is used to disperse the powder and to ensure that some of the dose is carried into the lungs [8]. To reach the small airways of the deep lung, the API particles in DPIs need to have an aerodynamic diameter of 1–5 μm to avoid impaction and particle sedimentation in the upper respiratory tract [9]. Consequently, the evaluation of API particle size is essential throughout all stages of pharmaceutical development of DPI products.

Conventional particle size analysis methods, such as laser diffraction, sieve analysis, and direct imaging techniques, cannot be applied, at least alone, to solid dosage forms due to the concomitant presence of excipients [10]. To the best of our knowledge, the literature data addressing the determination of API particle size and morphology within final pharmaceutical products are limited. Some researchers have developed methodologies that exploit the differences in melting points between excipients and APIs in tablet formulations. Using hot-stage microscopy, they successfully measured the particle size of tadalafil and meloxicam within pharmaceutical products [11]. This approach, although original, exhibits some limitations since the API melting point should be higher than the excipients. Finally, degradation and/or decomposition of the components is highly possible, resulting in toxicity or discoloration, etc.

Several studies employing micro-Raman chemical imaging have also been reported [12,13,14,15]. However, this technique alone appears to be more suitable for identifying the so-called “domain” particle size, as the discrimination of frequently occurring agglomerated particles remains unresolved. This technique has demonstrated considerable promise in the field and has therefore been employed in combination with other analytical methods to enable the measurement of API particle size in solid dosage forms.

Our team has developed a methodology for the determination of bismuth oxide API particle size in bismuth oxide 120 mg film-coated (FC) tablets. The sample preparation involved partial dissolution of excipients in selected solvents to enable efficient extraction of the API from the granules. Subsequent identification of particle size and morphology within the tablets was achieved using micro-Raman mapping spectroscopy in combination with ImageJ software (V.1.8.0) [16]. Similarly, Alexios Tsiligiannis et al. employed a micro-Raman spectrometer equipped with ParticleFinder™ module of LabSpec Software version 6.6.6.2 to chemically identify fluticasone propionate particles in Dymista^®^ nasal spray and to simultaneously assess their size distribution [17].

In recent years, a novel technique that has gained prominence for monitoring particle size and shape in solid dosage formulations is Morphologically Directed Raman Spectroscopy (MDRS^®^). MDRS integrates automated particle imaging with Raman microspectroscopy in a single platform, thereby enabling rapid, automated chemical and morphological characterization of individual components in multicomponent systems. According to the literature data, MDRS has been applied to determine the particle size of ibuprofen in a model formulation containing ibuprofen, starch, and lactose; to compare morphology and transparency properties of proteinaceous particles with reference standards; and to evaluate the particle size distribution of mometasone furoate monohydrate in five different nasal suspension drug products [18,19,20]. However, none of the aforementioned techniques have been applied to the determination of API particle size in DPIs, primarily due to the inherently low API concentration and the extremely small particle size characteristic of these formulations, since particulates must be ~2 µm or larger in size in order to be measured with MDRS [21].

The determination of API particle size in DPI formulations has previously been briefly reported [22,23]. Emily Landsperger et al. employed automated scanning electron microscopy (SEM) coupled with energy-dispersive X-ray (EDX) microanalysis. API identification was based on the presence of bromine, the characteristic element of the API, enabling its differentiation from the excipients [22]. In that study, particle size distribution was expressed using the *D*avg morphological parameter (average diameter). The instrument calculates this parameter by drawing sixteen chords through the center of each particle, with *D*avg representing the mean chord length. The average diameter was determined under the assumption of spherical particle geometry. However, the study was primarily illustrative, aiming to demonstrate the proposed method’s capability, and did not address any analytical issues, equivalence to established particle sizing techniques, or applicability to multiple commercial DPI products under low API loading conditions.

In the present study, we sought to expand upon this earlier work by establishing a controlled manual SEM–EDX analytical workflow and evaluating its quantitative performance, robustness (through APIs containing different characteristic elements), and limitations for API particle size determination in multiple finished DPI products with an API content as low as 0.1% *w*/*w*. We successfully determined the particle size and morphology of umeclidinium bromide and vilanterol trifenatate in Anoro^®^, fluticasone furoate and vilanterol trifenatate in Relvar^®^ and tiotropium bromide in Spiriva^®^. Particle size distribution was expressed in terms of d10, d50, and d90 values, analogous to the laser diffraction technique. The proposed approach can be implemented in pharmaceutical laboratories for bioequivalence studies with minor modifications.

## 2. Results

### 2.1. Samples Information

The information regarding the dosage form, the components and the % *w*/*w* content of the DPIs used for this study are presented in Table 1 below.

### 2.2. Developing the Methodology for the Identification and Calculation of API Particle Size in DPI Products

**Figure 1:**
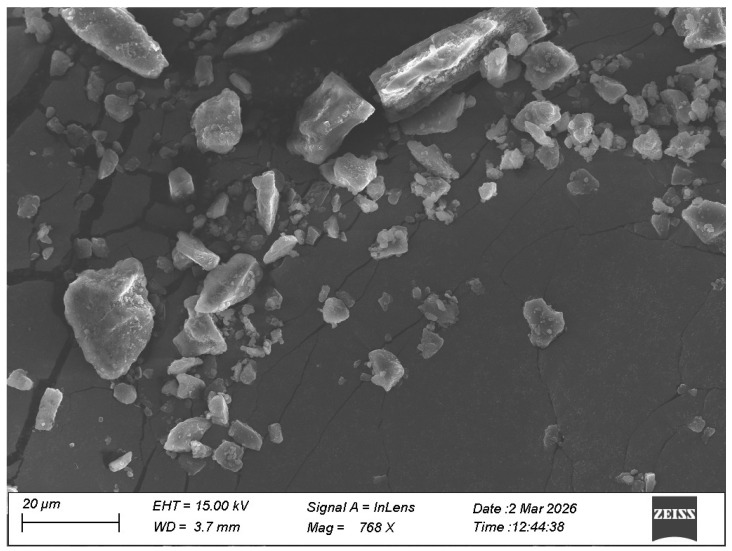
SEM micrograph of the first selected area of Spiriva^®^ 18 µg inhalation powder (hard capsule) at 768× magnification.

A principal challenge in the present study was the reliable identification of API particles within dry powder inhaler formulations, given that the APIs are micronized and present at very low concentrations (<1% *w*/*w*). Consequently, electron microscopy techniques—specifically scanning electron microscopy—were selected, as SEM is widely recommended for the characterization of micronized powders due to its ability to resolve surface features in the 1–10 nm range [29]. Initially, four to six relatively large fields of view were captured for each sample at magnifications ranging from approximately 200× to 1000×, with the aim of obtaining a representative sample area. An example of such an image is provided in Figure 1.

**Figure 2:**
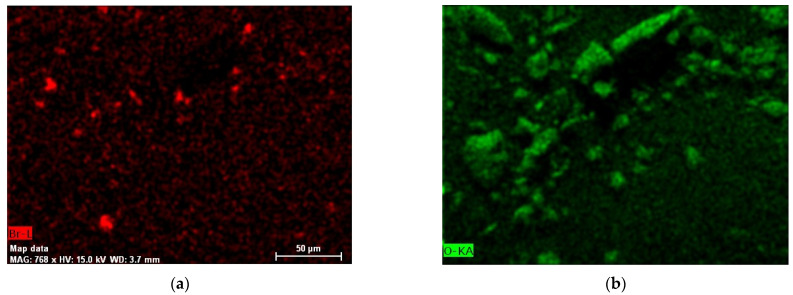
EDX elemental maps showing (**a**) bromide distribution, indicated by intense red pixels, and (**b**) oxygen distribution, indicated by intense green pixels, scale bar: 50 μm.

As illustrated in Figure 1, the predominant features were large, rectangular particles corresponding to lactose monohydrate. Numerous smaller particles were also visible; however, these could represent either API particles or lactose fragments. Thus, a subsequent step was required to discriminate between lactose and API particles. To achieve this, each area was subjected to elemental mapping using energy-dispersive X-ray microanalysis for 30 min. EDX enables the determination of elemental composition at the particle level, and each API contained at least one distinctive elemental marker that facilitated its identification (Figure 2).

As shown in Figure 2, the EDX elemental maps revealed red and green pixel clusters; the intense red pixels corresponded to regions containing the API (e.g., bromide, chlorine, or fluorine, depending on the formulation), while the green pixels indicated oxygen, characteristic of lactose.

**Figure 3:**
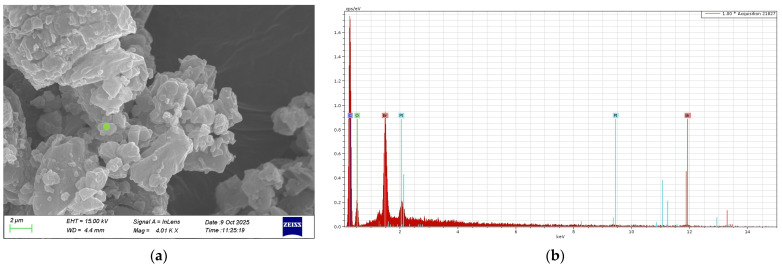
(**a**) Higher-magnification SEM image of a selected red pixel cluster from Figure 2a (marked with a green dot), acquired at 4.01 k× magnification; (**b**) corresponding EDX spectrum confirming bromide and, therefore, the presence of the API.

To confirm the presence of the API and assess the morphology of individual particles, each intense red pixel cluster was re-examined at higher magnification (Figure 3a). A focused EDX point analysis (“spot scan” in green dot of Figure 3a) was then conducted for 3 min to verify the presence of the API via its characteristic elemental peak (Figure 3b).

**Figure 4:**
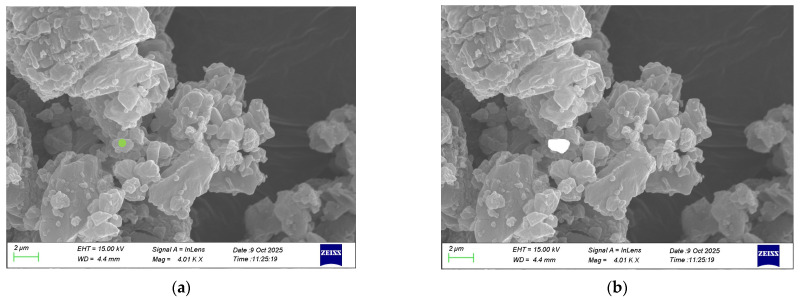
(**a**) Original SEM image of an identified API particle (green dot) and (**b**) the corresponding ImageJ (V.1.8.0) output after particle boundary tracing (white area), both at 4.01 k× magnification.

Following confirmation of particle identity, the particle size of each API particle was measured using ImageJ software (V.1.8.0). The area of each particle was delineated manually using the free-hand selection tool to trace its perimeter (white area in Figure 4b). ImageJ (V.1.8.0) subsequently computed the particle area and derived an equivalent particle diameter.

In the present study, approximately four to five independent SEM fields were randomly selected per formulation, and all API particles identified within each field were measured avoiding possible analyst bias concerns. Initially, 50 particles per formulation were analyzed, as reported in the manuscript. To evaluate method repeatability and the effect of increasing the number of measured API particles, additional measurements were performed by increasing the total number of analyzed particles to 100 and 150 in Spiriva® formulation. The relative standard deviation (%RSD) of particle size measurements (d10, d50 and d90) was calculated for each dataset (Table 2 and Table 3) and compared against the specifications available in pharmacopeia (Table 4) [30].

The results from Table 2 demonstrated that the %RSD remained within acceptable limits for all PSD values, and thus the method can be considered repeatable.

Increasing the particle count from 50 to 100 and 150 did not significantly reduce variability nor alter the calculated d10, d50, and d90 values (Table 3). These findings indicate that the variance observed was sufficiently low and that the analysis of 50 particles already provided statistically stable and representative particle size distribution parameters.

**Scheme 1:**
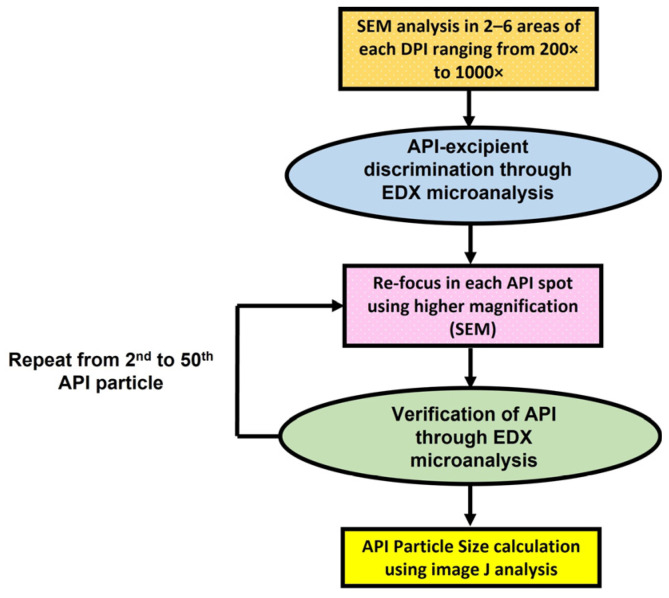
Flow chart describing the methodology developed for the identification and calculation of API particle size in DPI products.

The total duration of the experimental work and subsequent data analysis is approximately 15 h per formulation. The overall step by step procedure is described in Scheme 1.

### 2.3. Spiriva^®^ 18 Microgram Inhalation Powder, Hard Capsule

**Figure 5:**
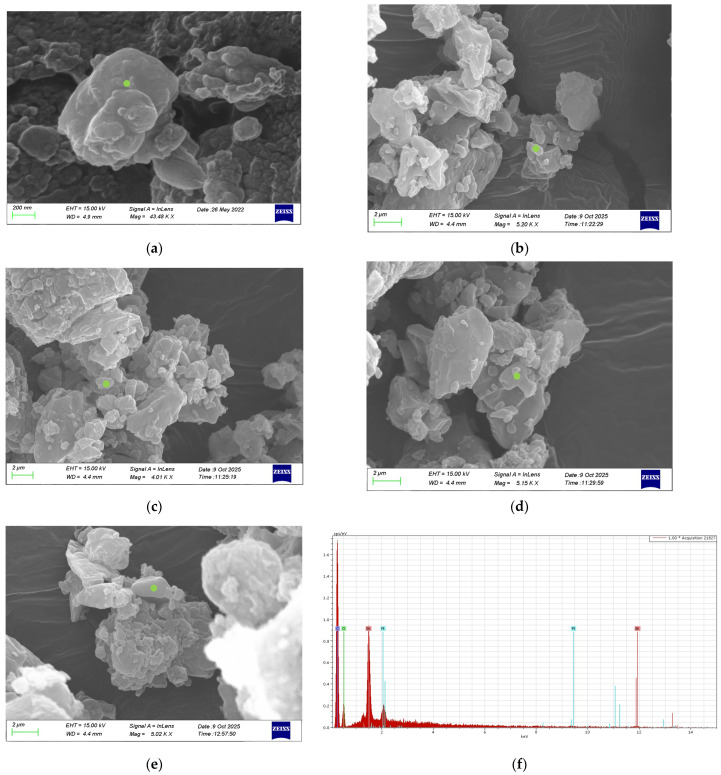
(**a**–**e**) Images of detected tiotropium bromide API particles (green dots) using SEM–EDX along with (**f**) the corresponding EDX spectrum confirming bromide and, therefore, the presence of the API.

The procedure described in Section 2.2 was first applied to the identification of tiotropium bromide API in Spiriva^®^ 18 µg inhalation powder (hard capsules). Four randomly selected regions of the sample were initially captured using SEM, and by following the full analytical workflow, approximately 50 API particles were identified and subsequently measured. For brevity, five representative API particles along with the relevant EDX graph are presented in Figure 5 (the rest are available upon request).

**Figure 6:**
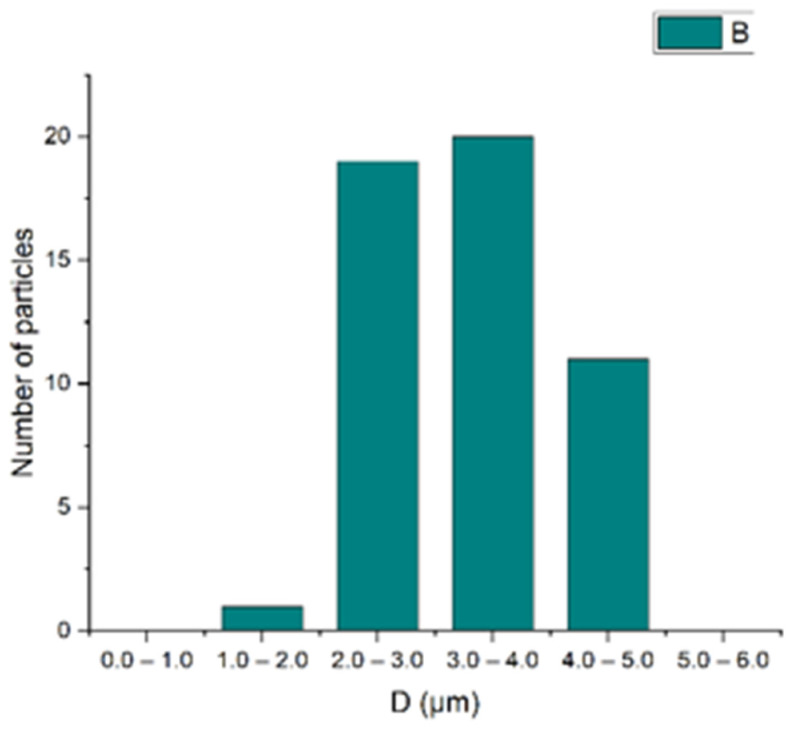
PSD histogram of detected tiotropium bromide API particles.

The identified tiotropium bromide API particles exhibited irregular morphology, predominantly displaying spherical or rectangular geometries. The particle size distribution of the API in Spiriva^®^ 18 µg inhalation powder (hard capsules) was determined as described in Section 2.2. Initially, the projected areas of the API particles were measured using ImageJ (V.1.8.0) software. Then, each API particle’s area was interpreted as that of an equivalent spherical particle, in accordance with the basic analytical principle applied in laser diffraction measurements. On this basis, the radius and diameter of each particle were calculated, and the corresponding particle volume was subsequently derived (available upon request). Finally, the cumulative volume distribution was used to determine the d(10), d(50), and d(90) particle size values and respective number-based histogram (Figure 6, Table 5).

The mean diameter along with the PSD values of tiotropium API in Spiriva^®^ 18 µg inhalation powder was successfully calculated and appeared to be micronized as expected [9]. The PSD histogram confirm that the PSDs of the analyzed APIs are predominantly unimodal with no evidence of secondary particle populations or pronounced long tails. This observation supports the validity of the percentile-based representation and further strengthens the interpretation of the results.

### 2.4. Anoro^®^ Ellipta 55 Micrograms/22 Micrograms Inhalation Powder

Anoro^®^ Ellipta 55 micrograms/22 micrograms inhalation powder comprises two double-foil blister strips. The first blister strip consists of umeclinidium Br, MgST, and lactose while the second contains vilanterol, MgST, and lactose.

#### 2.4.1. Umeclinidium Br Blister Strip

**Figure 7:**
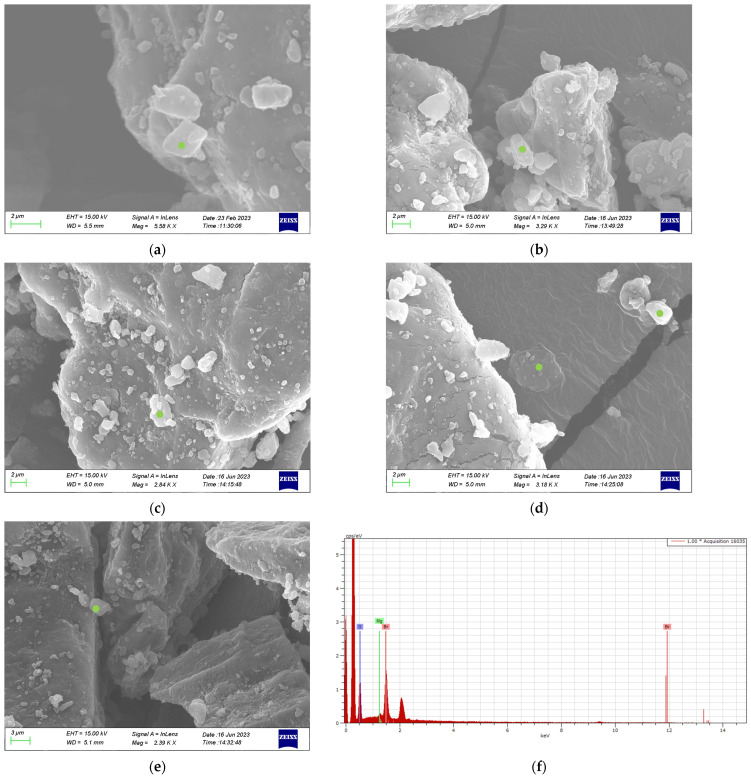
(**a**–**e**) Images of detected umeclinidium bromide API particles (green dots) using SEM–EDX along with (**f**) the corresponding EDX spectrum confirming bromide and, therefore, the presence of the API.

The same procedure as in the previous section was applied for the identification of umeclinidium bromide API in Anoro^®^ Ellipta inhalation powder. Bromide was the unique API element that was used to discriminate umeclinidium API in the current DPI. Similarly, two randomly selected regions of the sample were initially captured using SEM and scanned with EDX which led to the identification of approximately 50 API particles. Six representative API particles of those are presented in Figure 7 (the rest are available upon request).

**Figure 8:**
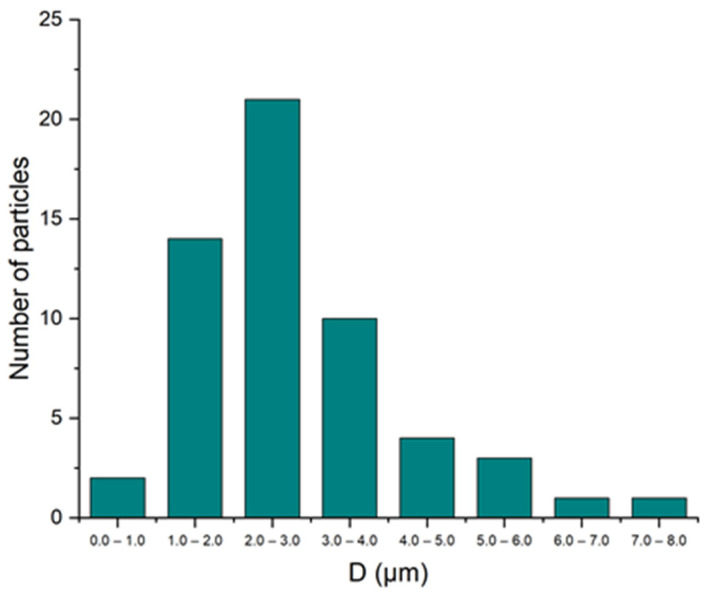
PSD histogram of detected umeclinidium bromide API particles.

The identified umeclinidium bromide API particles mostly exhibited the spherical morphology of a smooth surface. The particle size distribution of umeclinidium bromide API in Anoro^®^ Ellipta inhalation powder was determined as described in Section 2.2 (Figure 8, Table 6).

The mean diameter along with the PSD values of umeclinidium bromide API in Anoro^®^ Ellipta 55 micrograms/22 micrograms inhalation powder was successfully calculated and appeared to be micronized as expected [9]. The PSD histogram confirms that the PSDs of the analyzed APIs are predominantly unimodal with no evidence of secondary particle populations or pronounced long tails. This observation supports the validity of the percentile-based representation.

#### 2.4.2. Vilanterol Trifenatate Blister Strip (Anoro® Ellipta Inhalation Powder)

**Figure 9:**
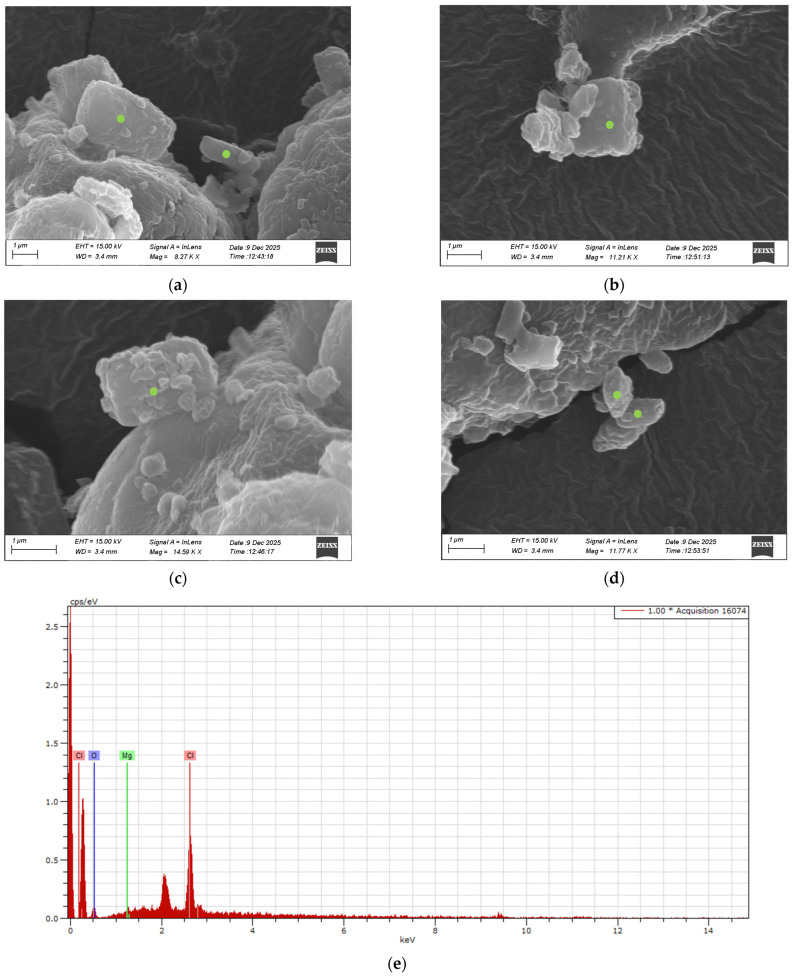
(**a**–**d**) Images of detected vilanterol trifenatate API particles of Anoro^®^ blister strip (green dots) using SEM–EDX along with (**e**) the corresponding EDX spectrum confirming chlorine and, therefore, the presence of the API.

The same procedure as in the previous section was applied for the identification of vilanterol trifenatate API in Anoro^®^ Ellipta inhalation powder. Chlorine was the unique API element that was used to discriminate vilanterol API in the current DPI. Following the same procedure as before, the identification of approximately 50 API particles was performed. Six representative API particles of those are presented in Figure 9 (the rest are available upon request).

**Figure 10:**
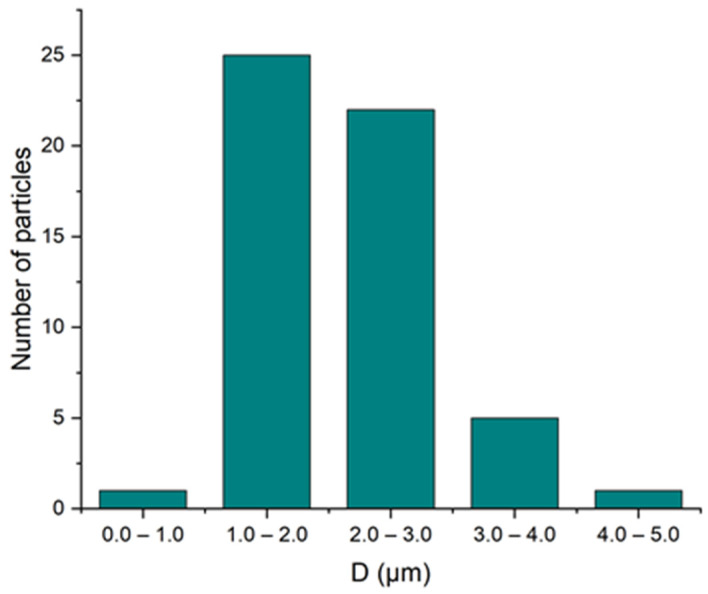
PSD histogram of detected vilanterol trifenatate API particles.

The identified vilanterol trifenatate API particles exhibited mostly columnar morphology. The particle size distribution of vilanterol trifenatate API in Anoro^®^ Ellipta inhalation powder was determined as described in Section 2.2 (Figure 10, Table 7).

The mean diameter along with the PSD values of vilanterol trifenatate API in Anoro^®^ Ellipta 55 micrograms/22 micrograms inhalation powder was successfully calculated and appeared to be micronized as expected [9]. The PSD histogram confirm that the PSDs of the analyzed APIs are predominantly unimodal with no evidence of secondary particle populations or pronounced long tails. This observation supports the validity of the percentile-based representation.

### 2.5. Relvar^®^ Ellipta 92 Micrograms/22 Micrograms Inhalation Powder

Relvar^®^ Ellipta 92 micrograms/22 micrograms inhalation powder comprises two double-foil blister strips. The first blister strip consists of fluticasone furonate and lactose while the second contains vilanterol, MgST, and lactose.

#### 2.5.1. Fluticasone Furonate Blister Strip

**Figure 11:**
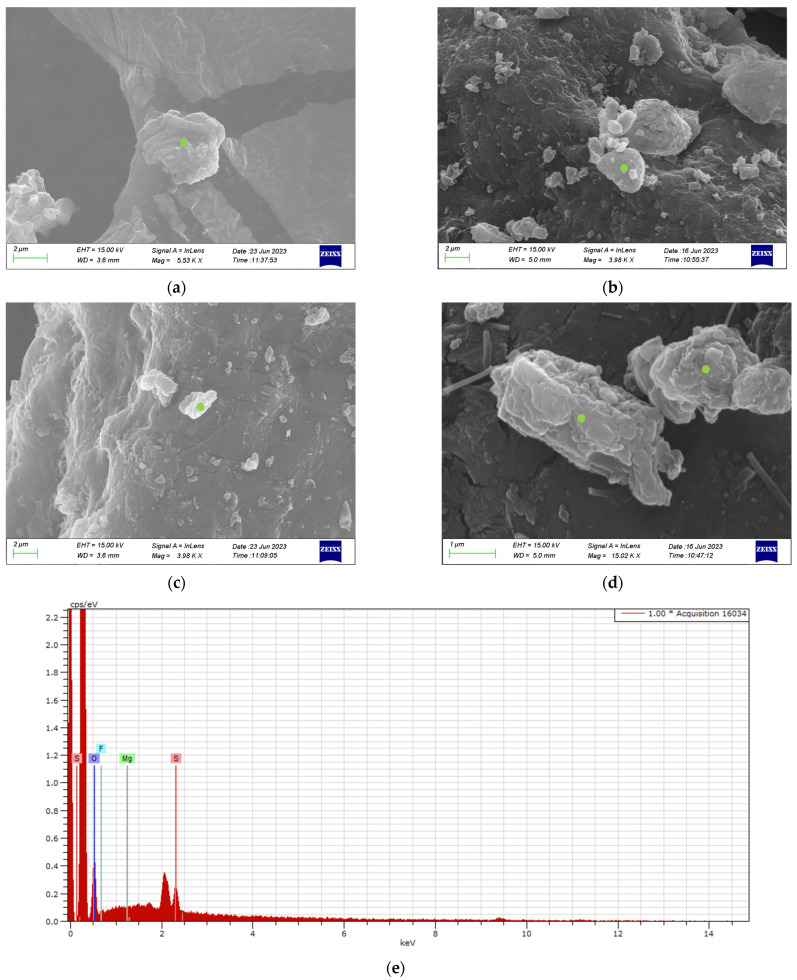
(**a**–**d**) Images of detected fluticasone furonate API particles (green dots) using SEM–EDX along with (**e**) the corresponding EDX spectrum confirming sulfur and, therefore, the presence of the API.

The same procedure as in the previous section was applied for the identification of fluticasone furonate API in Relvar^®^ Ellipta inhalation powder. Sulfur was the unique API element that was used to discriminate fluticasone furonate API in the current DPI. Similarly, three randomly selected regions of the sample were initially captured using SEM and scanned with EDX, which led to the identification of approximately 50 API particles. Five representative API particles of those are presented in Figure 11 (the rest are available upon request).

**Figure 12:**
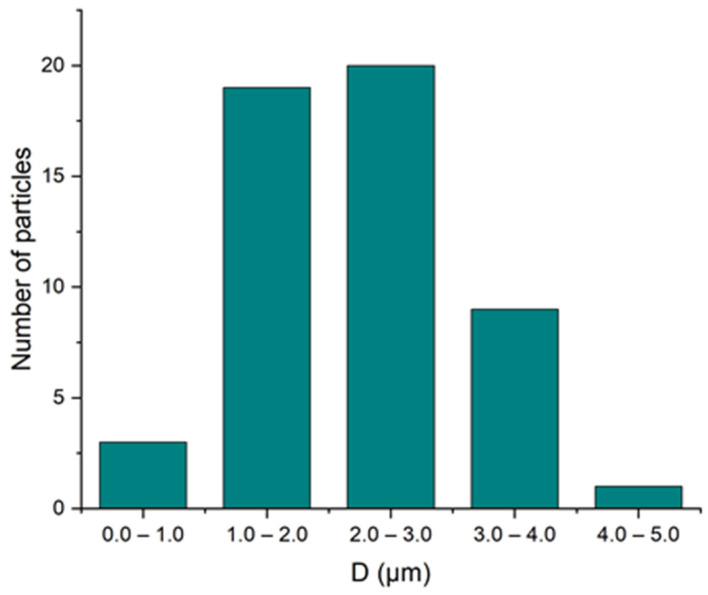
PSD histogram of detected fluticasone furonate API particles.

The identified fluticasone furonate API particles exhibited irregular shape (mostly spherical and columnar morphology) of rough (rugose) surface. Particles appeared more fused compared to the previous APIs. The particle size distribution of fluticasone furonate API in Relvar^®^ Ellipta inhalation powder was determined as described in Section 2.2 (Figure 12, Table 8).

The mean diameter along with the PSD values of fluticasone furonate API in Relvar^®^ Ellipta 92 micrograms/22 micrograms inhalation powder was successfully calculated and appeared to be micronized as expected [9]. The PSD histogram confirm that the PSDs of the analyzed APIs are predominantly unimodal with no evidence of secondary particle populations or pronounced long tails. This observation supports the validity of the percentile-based representation.

#### 2.5.2. Vilanterol Trifenatate Blister Strip (Relvar^®^ Ellipta Inhalation Powder)

**Figure 13:**
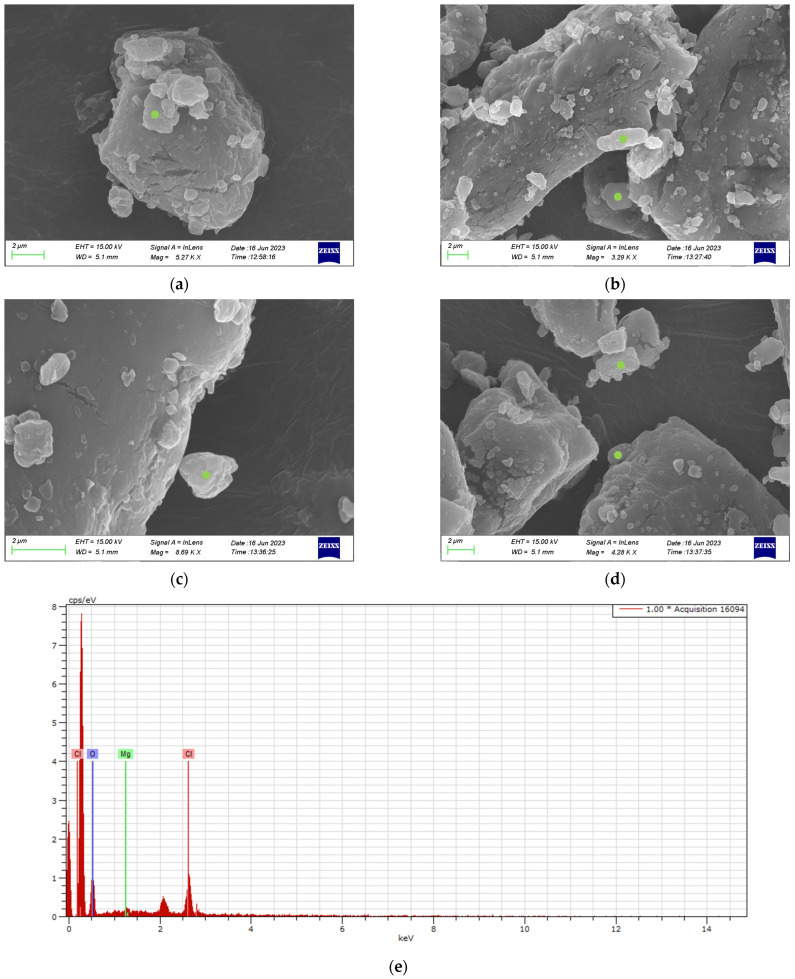
(**a**–**d**) Images of detected vilanterol trifenatate API particles of Relvar^®^ (green dots) using SEM–EDX along with (**e**) the corresponding EDX spectrum confirming chlorine and, therefore, the presence of the API.

As previously mentioned in Section 2.4.2., chlorine was the unique API element that was used for the identification of vilanterol trifenatate API in Relvar^®^ Ellipta inhalation powder. Following the same procedure as before, three randomly selected regions of the sample were initially captured using SEM and scanned with EDX, which led to the identification of approximately 50 API particles. Six representative API particles of those are presented in Figure 13 (the rest are available upon request).

**Figure 14:**
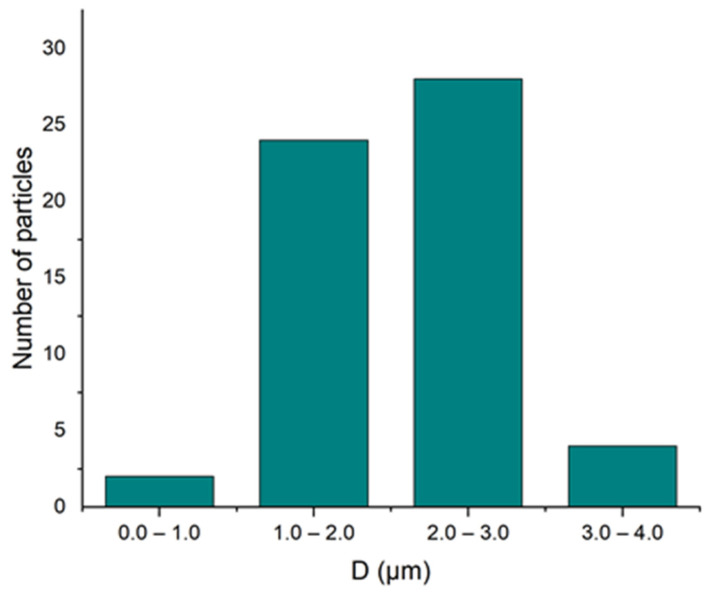
PSD histogram of vilanterol trifenatate API in Relvar^®^ Ellipta 92 micrograms/22 micrograms inhalation powder.

The identified vilanterol trifenatate API particles mostly exhibited the columnar and spherical morphology of a smooth surface. The particle size distribution of vilanterol trifenatate API in Relvar^®^ Ellipta inhalation powder was also determined as described in Section 2.2 (Figure 14, Table 9).

The mean diameter along with the PSD values of vilanterol trifenatate API in Relvar^®^ Ellipta 92 micrograms/22 micrograms inhalation powder was successfully calculated and appeared to be micronized as expected [9]. The PSD histogram confirm that the PSDs of the analyzed APIs are predominantly unimodal with no evidence of secondary particle populations or pronounced long tails. This observation supports the validity of the percentile-based representation.

### 2.6. Cross-Validation of PSD Measurements Using Laser Diffraction

**Figure 15:**
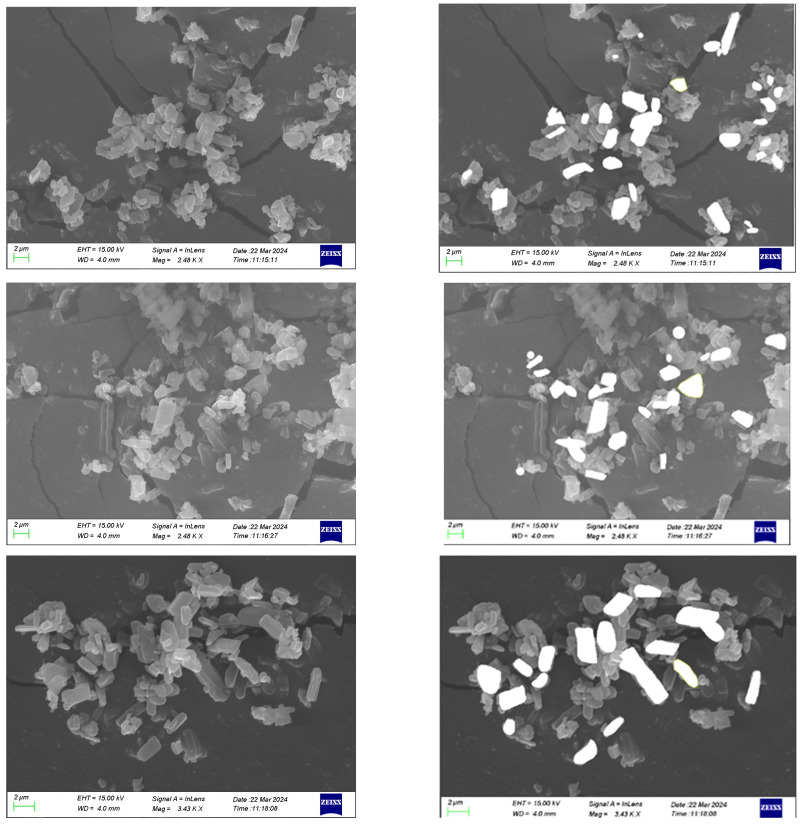
Images of vilanterol trifenatate API Lot: P-37 in original mode (**left**) and after particle boundary tracing (white area) using ImageJ (V.1.8.0) analysis method (**right**) using SEM.

**Figure 16:**
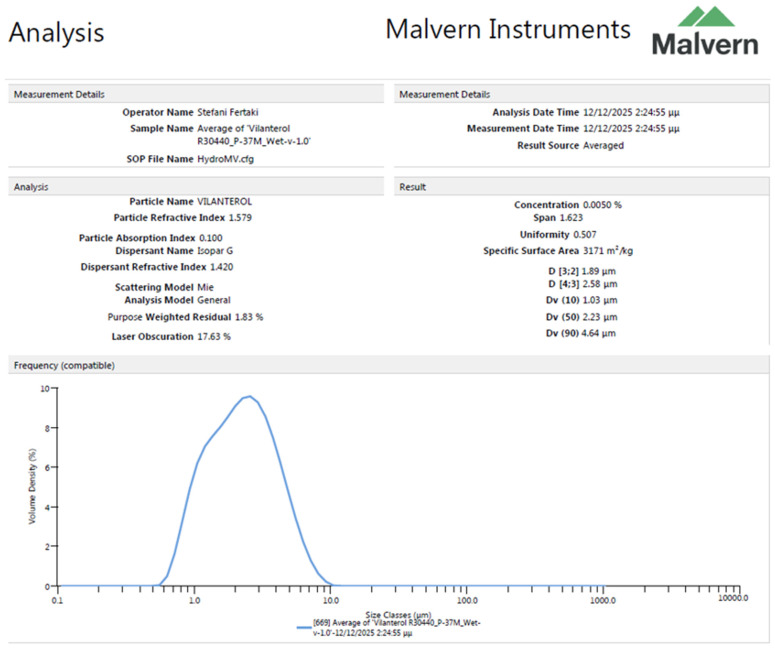
PSD histogram of vilanterol trifenatate API Lot: P-37 using laser diffraction.

To assess the reliability of the SEM–EDX methodology developed for API particle size distribution (PSD) determination in DPIs, the developed approach was applied to pure vilanterol API (Figure 15) and the resulting PSD values were cross-validated using a laser diffraction method (Figure 16, Table 10). The laser diffraction procedure employed for comparison was provided by the API manufacturer (PSD histogram available upon request).

Based on the data presented in Table 10 and in Figure 15 and Figure 16 and in accordance with ICH guidelines [31], the SEM–EDX-derived PSD values demonstrate overall consistency with those obtained by laser diffraction. While some variability was observed for the lower and upper percentile values (d10 and d90), the median particle size (d50) showed close agreement between the two techniques. The relatively higher relative standard deviations observed for d10 and d90 likely reflect the sensitivity of distribution tails to sampling size and methodological differences between area-based image analysis and volume-based laser diffraction measurements. In contrast, the central percentile (d50), which is less influenced by distribution extremes, demonstrated strong agreement between techniques. Given that the comparison was performed on pure API samples rather than finished formulations, this assessment should be considered supportive rather than direct validation of formulation-level equivalence.

## 3. Discussion

The identification of particle size and morphology of individual components in pharmaceutical formulations is of critical importance, as these parameters are known to significantly influence the dissolution rate of the API, bioavailability, and bioequivalence. This is particularly relevant for dry powder inhalers, where accurate determination of API particle size within the formulation is essential due to the requirement for particles to reach the human lungs and achieve therapeutic efficacy. Commonly used particle size analysis techniques were not applicable to the formulations under investigation due to the simultaneous presence of excipients and the extremely low API content.

For these reasons, in the present study, a methodology was developed to successfully identify and characterize the particle size and morphology of tiotropium bromide API in Spiriva^®^ 18 μg inhalation powder (hard capsules), umeclidinium bromide API and vilanterol trifenatate API in Anoro^®^ Ellipta inhalation powder, and fluticasone furoate API and vilanterol trifenatate API in Relvar^®^ Ellipta inhalation powder.

Given the low API content and the expected micron-scale particle size, the experimental approach was based on a combination of electron microscopy techniques, specifically scanning electron microscopy, coupled with elemental microanalysis using energy-dispersive X-ray spectroscopy. The identification of each API was achieved by exploiting the presence of a unique elemental marker specific to the active ingredient, enabling differentiation from the excipient matrix during large-area EDX scans. Following initial identification, each region of interest was examined at higher magnification, and the API particle was further confirmed by repeated microanalysis.

This procedure was systematically repeated until a minimum of 50 API particles per formulation were identified and measured. Particle diameters and the corresponding particle size distribution parameters (d10, d50, and d90) were determined using ImageJ (V.1.8.0) software. Particle areas were measured using free-hand selection and converted to equivalent spherical diameters based on area equivalence. The same analytical procedure was applied to pure API samples, and the resulting PSD values were compared with those obtained using laser diffraction, a commonly employed technique for particle size analysis. Statistical evaluation demonstrated overall consistency between the two methods, thereby confirming the validity and robustness of the developed methodology.

While additional morphometric descriptors such as aspect ratio or Feret diameters may provide further insight into particle anisotropy, the primary objective of the present study was the determination of regulatory-relevant PSD parameters (d10, d50, d90). These parameters are commonly employed in FDA- and ICH-aligned specifications for particle size control. The developed methodology can, however, be readily extended to include additional shape descriptors where detailed morphological characterization is required.

All APIs in the evaluated DPI products were found to be micronized, as expected for inhalation formulations, while particle morphology varied among the samples, ranging from predominantly spherical to columnar particles with either smooth or rugose surfaces.

The present methodology provides a direct analytical pathway for determining API particle size distribution within intact DPI formulations without prior API isolation or excipient removal. From a theoretical perspective, this enables true component-resolved particle size analysis in multicomponent systems with extremely low API loading.

From a practical standpoint, the method offers significant utility for pharmaceutical development and regulatory evaluation. It enables reverse engineering of commercial DPI products, supports bioequivalence investigations, and provides a potential quality control tool for verifying API micronization within finished dosage forms. Importantly, the methodology is applicable under low API content conditions (<1% *w*/*w*), which are typical for inhalation therapies and remain analytically challenging using conventional particle sizing approaches.

The developed method may be implemented in pharmaceutical research and quality control as a routine approach for the determination of API particle size and morphology in DPI products.

Limitations: The applicability of the SEM–EDX approach further depends on the presence of a distinctive elemental marker in the API. APIs composed predominantly of light elements may exhibit reduced elemental contrast due to low X-ray emission intensity or spectral overlap. In such cases, increasing EDX acquisition time can improve counting statistics and signal-to-noise ratio, although spatial resolution remains constrained by electron interaction volume, particularly for submicron particles. Additionally, SEM imaging inherently involves field selection, which may introduce sampling bias despite the analysis of multiple fields and the measurement of all identifiable particles within each field. Manual particle delineation may also introduce operator-dependent variability, particularly for irregular or partially overlapping particles. To minimize this effect, multiple fields were systematically analyzed and all chemically identified API particles within each field were measured. Increasing particle count enhances statistical robustness and reduces variability, especially for distribution tails.

## 4. Materials and Methods

### 4.1. Samples

The monohydrate tiotropium bromide (Cipla limited, Mumbai, India), monohydrate α-lactose (DFE Pharma, Goch, Germany), magnesium stearate (Peter Greven Nederland C V, Netherlands), umeclidinium bromide (INKE Pharmaceutical, Barcelona, Spain), vilanterol trifenatate (Glaxosmithkline pharmaceuticals, Mumbai, India) fluticasone furoate (Sterling S.p.A., Deeside, Wales, UK) and glycopyrronium bromide (INKE Pharmaceutical, Barcelona, Spain) were kindly provided by ELPEN S.A. (Pikermi, Attica, Greece). The Spiriva^®^ 18 microgram inhalation powder (Boehringer Ingelheim, Biberach, Germany), Anoro^®^ 55 micrograms/22 micrograms inhalation powder (Glaxosmithkline pharmaceuticals, London, UK) and Relvar^®^ 92 micrograms/22 micrograms inhalation powder (Glaxosmithkline pharmaceuticals, Dublin, Ireland) were purchased from a local pharmacy store.

### 4.2. Scanning Electron Microscopy (SEM)

The electron microscope used in the present work was the SUPRA 35VP from Zeiss, (Carl Zeiss AG, Oberkochen, Germany) equipped with the SmartSEM User Interface software (v.5.0). Images were taken at 15 kV using the Inlens-SE detector, which captures secondary electrons. The samples were surface-coated with gold or gold/palladium using a SCD 004 sputter coater (BAL-TEC AG, Balzers, Liechtenstein)) with an argon gas atmosphere for 3 min and a current intensity of 13 mA before measurement.

### 4.3. Energy-Dispersive X-Ray Microanalysis (EDX)

The energy-dispersive spectrometer (EDS) was the QUANTAX EDS with an XFlash^®^ 7 detector and Esprit 1.8 software (Bruker Nano GmbH, Berlin, Germany). The measuring time of the mapped areas was 1200 s (10,000 cycles). The point time was 2 s of 10,000 counts. The analysis region was from 0.25 keV to 10.00 keV while fast quantification was selected.

### 4.4. ImageJ

ImageJ (V.1.8.0) software was used for the calculation of API particle size. The area of each particle was automatically calculated by drawing each particle perimeter using free-hand selection. The diameter of each particle was then calculated from the initially measured area when considered as a sphere.

### 4.5. Laser Diffraction

A laser analyzer Mastersizer 3000 (Malvern Instruments, Malvern, UK) with a Hydro MV accessory was used to determine the particle size distribution (PSD) of the samples. The measurement range of the apparatus is 0.02–2000 µm. A red light of 633 nm wavelength was the source. The stirrer speed was set at 3000 rpm, the obscuration range was set at 10–30%, and the particle size was calculated on a volume basis using the Mie theory. Isopar G and span 85 were used as the dispersant and surfactant, respectively. The API powder was added directly in the cell and the slurry was sonicated for 3 min internally.

## 5. Conclusions

In the present study, a controlled SEM–EDX analytical workflow was developed and applied for the direct identification and particle size determination of active pharmaceutical ingredients within finished dry powder inhaler (DPI) formulations containing less than 1% *w*/*w* API. The methodology combined large-area elemental mapping for selective API localization with high-magnification imaging and confirmatory spot microanalysis, followed by manual image-based measurement and the calculation of equivalent spherical diameters. Particle size distribution parameters (d10, d50, and d90) were determined for multiple commercial DPI products, including Spiriva^®^, Anoro^®^ Ellipta, and Relvar^®^ Ellipta.

The approach enabled chemically selective discrimination of APIs within complex excipient matrices without prior formulation modification or API extraction. Cross-validation against laser diffraction measurements of pure API material demonstrated overall consistency between the two techniques, supporting the reliability of the developed SEM–EDX methodology within its defined analytical framework. All evaluated APIs were confirmed to be appropriately micronized for pulmonary delivery, while exhibiting distinct morphological characteristics across products.

In addition to its analytical robustness, the proposed SEM–EDX workflow offers both theoretical and industrial value. The methodology enables chemically selective particle size determination of APIs within finished DPI formulations, even at very low drug loading levels, without formulation modification. This capability may support generic drug development, regulatory assessment of inhalation products, bioequivalence studies, and quality control monitoring of critical particle size attributes in DPI manufacturing.

## Figures and Tables

**Table 1:** Composition and dosage form of the commercial DPI products evaluated in the present study.

Product	Components	Content (% *w*/*w*)	Dosage Form
Spiriva^®^ [24]	Tiotropium bromide (Br) monohydrate	~0.4% *w*/*w*	Hard capsules
	Lactose monohydrate	~99.6% *w*/*w*	
Anoro^®^ [25,26]	Umeclidinium bromide	~0.3% *w*/*w*	2 double-foil blister strips ^1^
	Vilanterol trifenatate	~0.16% *w*/*w*	
	Magnesium stearate (MgST)	~0.8% *w*/*w*	
	Lactose monohydrate	~98.7% *w*/*w*	
Relvar^®^ [27,28]	Fluticasone furonate	~0.4% *w*/*w*	2 double-foil blister strips ^2^
	Vilanterol trifenatate	~0.1% *w*/*w*	
	Magnesium stearate	~0.5% *w*/*w*	
	Lactose monohydrate	~99% *w*/*w*	

^1^ The first blister strip consists of umeclinidium Br, MgST, and lactose while the 2nd strip consists of vilanterol, MgST, and lactose. ^2^ The first blister strip consists of fluticasone and lactose while the 2nd strip consists of vilanterol, MgST, and lactose.

**Table 2:** Repeatability.

Repeat	No. of Total API Particles Measured	d(0.1)	d(0.5)	d(0.9)
A	51	2.20	3.20	4.50
B	53	1.62	2.60	4.20
C	46	1.60	2.45	3.40
Mean		1.81	2.75	4.03
SD		0.34	0.40	0.57
% RSD		18.86	14.43	14.10

**Table 3:** Effect of increasing the number of measured API particles on PSD parameters and statistical variability.

	Number of Total Tiotropium Br API Particles Measured	d(0.1)	d(0.5)	d(0.9)
	51	2.20	3.20	4.50
	97	2.30	3.24	4.50
	150	1.97	3.10	4.50
Mean		2.16	3.18	4.50
SD		0.17	0.07	0.00
% RSD		7.85	2.27	0.00

**Table 4:** Acceptance criteria for the repeatability and robustness test according to Ph. Eur. 9.2 chapter 2.9.31 [30].

Volume Percentile	Acceptance Criteria	
	Result ^1^ ≥ 10 μm	Result ^1^ < 10 μm
D(0.1) (µm)	RSD max. 15%	RSD max. 30%
D(0.5) (µm)	RSD max. 10%	RSD max. 20%
D(0.9) (µm)	RSD max. 15%	RSD max. 30%

^1^ Refers to the overall average of all measurements performed in the repeatability and robustness test.

**Table 5:** Mean diameter and cumulative particle size distribution (PSD) values of tiotropium bromide API in Spiriva^®^ 18 µg inhalation powder (hard capsules), calculated from a total of 51 identified API particles.

Mean Diameter	D[0.1]	D[0.5]	D[0.9]
2.36 µm	2.2 µm	3.2 µm	4.5 µm

**Table 6:** Mean diameter and cumulative particle size distribution (PSD) values of umeclinidium bromide API in Anoro^®^ Ellipta 55 micrograms/22 micrograms inhalation powder, calculated from a total of 56 identified API particles.

Mean Diameter	D[0.1]	D[0.5]	D[0.9]
2.91 µm	2.75 µm	4.6 µm	7.3 µm

**Table 7:** Mean diameter and cumulative particle size distribution (PSD) values of vilanterol trifenatate API in Anoro^®^ Ellipta 55 micrograms/22 micrograms inhalation powder, calculated from a total of 54 identified API particles.

Mean Diameter	D[0.1]	D[0.5]	D[0.9]
2.07 µm	1.91 µm	2.8 µm	4.3 µm

**Table 8:** Mean diameter and cumulative particle size distribution (PSD) values of fluticasone furonate API in Relvar^®^ Ellipta 92 micrograms/22 micrograms inhalation powder, calculated from a total of 52 identified API particles.

Mean Diameter	D[0.1]	D[0.5]	D[0.9]
2.25 µm	2.03 µm	3.3 µm	3.98 µm

**Table 9:** Mean diameter and cumulative particle size distribution (PSD) values of vilanterol trifenatate API in Relvar^®^ Ellipta 92 micrograms/22 micrograms inhalation powder, calculated from a total of 58 identified API particles.

Mean Diameter	D[0.1]	D[0.5]	D[0.9]
2.14 µm	1.83 µm	2.6 µm	3.6 µm

**Table 10:** PSD values of vilanterol trifenatate API Lot: P-37 using SEM–EDX and laser diffraction.

Sample Information	D[0.1]	D[0.5]	D[0.9]
Vilanterol trifenatate API Lot: P-37 using SEM–EDX	1.58 um	2.36 um	3.10 um
Vilanterol trifenatate API Lot: P-37 using laser diffraction	1.03 µm	2.23 µm	4.64 µm
RSD%	29.8	4.0	28.1

## Data Availability

The original contributions presented in this study are included in the article. Further inquiries can be directed to the corresponding author.

## Abbreviations

The following abbreviations are used in this manuscript:

API	Active Pharmaceutical Ingredient
DPI	Dry Powder Inhaler
SEM	Scanning Electron Microscopy
EDX	Energy-Dispersive X-ray Microanalysis
FC	Film-Coated
MDRS	Morphologically Directed Raman Spectroscopy
MgST	Magnesium Stearate
PSD	Particle Size Distribution
ICH	International Council for Harmonization of Technical Requirements of Pharmaceuticals for Human Use
